# Trends of Malaria Prevalence in Selected Districts of Kaffa Zone, Southwest Ethiopia

**DOI:** 10.1155/2022/3556140

**Published:** 2022-10-14

**Authors:** Tadesse Duguma, Eyob Tekalign, Mitiku Abera

**Affiliations:** Department of Medical Laboratory Science, College of Health Science and Medicine, Mizan-Tepi University, Mizan-Aman, Ethiopia

## Abstract

**Background:**

Malaria remains one of the world's major public health issues, particularly in low- and middle-income countries. In Ethiopia, cases have declined over the last decade, and attempts to eradicate the illness are underway. The purpose of this study was to determine trends in malaria prevalence in selected areas of the Kaffa zone during the last five years (Decha and Gimbo districts).

**Methods:**

All malaria cases registered from 2017 to 2021 were reviewed to assess the trends of malaria prevalence. A checklist was used to collect the secondary data from registers and entered it into an Excel spreadsheet, which was then exported to the Statistical Package for the Social Sciences (SPSS) version 25.0 for analysis. The malaria incidence rate was calculated with the total number of person-years as the denominator and the number of new cases as the numerator. Seasons, years, gender, age, and malaria parasite species were all used to show trends in malaria transmission in the areas. Tables and figures were used to present the data.

**Results:**

Out of 20,045 individuals screened for malaria, 13.6% (2,732/20,045) of them were recorded to have *Plasmodium* species tested using microscopy and rapid diagnostic test (RDT). *Plasmodium falciparum, Plasmodium vivax*, and mixed infections (*Plasmodium falciparum* + *Plasmodium vivax*) accounted for 1200 (5.9%), 1114 (5.56%), and 418 (2.09%) of the confirmed malaria cases, respectively. Overall, malaria incidence decreased with an interannual variation, from 4.08 cases per 1000 person-years in 2017 to 3.62 cases per 1000 person-years in 2018, then increased to 4.94 cases per 1000 person-years in 2021.

**Conclusion:**

The malaria trend over the last five years has revealed a nonconsistent pattern of cases in different years. The number of malaria cases has shown an increase over the period of 2017 to 2021. Interannual and intra-annual variations have been observed in the transmission of the disease.

## 1. Introduction

### 1.1. Background

Malaria is one of the most serious and widespread human diseases. A total of 627,000 malaria deaths were reported in 2021, which is also evidenced by an increase in the cases from the previous two years; 2019 and 2020, which accounts for approximately 95% of cases, with WHO African region countries accounting for the majority of this increase. The total malaria deaths in children under the age of five years showed a reduction from 87% in 2000 to 77% in 2020 [1]. Globally, an estimated 219 million cases of malaria were reported in 2017, with 435,000 deaths recorded compared to 451,000 in 2016 and 607,000 in 2010 [2].

According to the 2018 global malaria report, the global malaria incidence rate has decreased by 18% from 2010 to 2017. Similarly, during the same period, the estimated number of cases decline from 239 million to 219 million, and the number of deaths fall from 607,000 to 435,000. Despite this, malaria continues to be a global public health issue [2–4]. Almost half of the world's countries are now malaria-free as a result of integrated malaria-fighting efforts over the last decade and a half [5]. Malaria continues to be a major cause of morbidity and death in many parts of the world. It has negative socioeconomic consequences in addition to its health effects [6]. Human malaria is mainly caused by four species of parasites in the genus *Plasmodium: Plasmodium falciparum, Plasmodium vivax, Plasmodium ovale*, and *Plasmodium malariae*. *Plasmodium knowlesi*, which has a limited geographical distribution, also infects humans [7, 8].

Ethiopia is one of Africa's top five countries with large population suffering a lot from malaria. Despite the country's long history of malaria eradication and control, which dates back to the 1950s, the disease continues to pose a significant threat [9].

Malaria transmission in Ethiopia is spatiotemporally dynamic, with unstable and seasonal transmission in the majority of the country's malaria-endemic areas. Perennial transmission occurs in the country's western lowlands, where conditions are conducive to malaria transmission all year [10]. Environmental and climatic factors, such as altitude and rainfall, play a major role in transmission. The two common forms of *Plasmodium* species in Ethiopia are *P. falciparum* and *P. vivax* which are coendemic in the country [11, 12].

About 60% of the population, an estimated 60 million people live in these malaria-risk areas [13, 14]. In Ethiopia, there were 904,495 malaria cases and 213 deaths in 2018. *Plasmodium falciparum* caused the most morbidity, with 724,996 (80.1%), followed by *P. vivax* (18.4%) and mixed infections, with 166,340 (18.4%) and 13,159 (1.5%), respectively. Malaria affects people from all walks of life, with children bearing the most of the burden, followed by pregnant women [15, 16].

Ethiopia has implemented various malaria prevention and control strategies to reduce malaria morbidity, mortality, and economic loss, recognizing the disease's burden. Distribution of insecticide-treated bed nets, indoor residual spraying, drainage of stagnant water, and improved healthcare utilization are important in the fight against the disease. Additionally, intermittent preventive therapy for pregnant women, early diagnosis, prompt treatment, prevention, rapid management of malaria epidemics, and disease surveillance were among the interventions used. Malaria prevention and control services have been provided free of charge in the country. Ethiopia is currently working on a malaria elimination program that aims to eradicate the disease by 2030 [17, 18].

The malaria control strategies that have been implemented consistently around the world have yielded notable results, and Ethiopia is one of the countries that has made significant progress. In Ethiopia, the disease's incidence, prevalence, and mortality rates all followed a similar pattern [15, 19–22]. The trends analysis of malaria prevalence in all malariaious areas could help in the understanding of disease transmission dynamics.

Evidence-based and area-specific interventions require this type of information. As a result, the objective of this study was to assess the malaria transmission trends over the last five years in the Decha and Gimbo districts of Southwest Ethiopia. The districts' altitudes range from 1,000 to 3,350 meters above sea level, with mean annual rainfall ranging from 1001 to 2200 millimeters for January (the least) and May (the most) and mean annual temperatures ranging from 10.1 to 27.5 degrees Celsius [23]. The two districts had a combined population of 218,779 people at the time of the last census. The districts are divided into 78 kebeles (the lowest administrative unit). The community is served by public and private health care facilities.

In the last decade, as vector control interventions such as indoor residual spray (IRS) and long-lasting insecticidal nets (LLINs) have become more widely used for malaria control, the burden of malaria has dramatically decreased in several areas of Ethiopia. These vector control interventions are likely to have played a critical role in malaria control [24, 25]. Determining the trends of malaria infections in the study areas, which have been malariaious historically, is therefore essential in designing appropriate malaria control and eventual elimination strategies.

## 2. Materials and Methods

### 2.1. Study Area and Period

The study involved using secondary data (2017-2021) from health facilities in the Decha and Gimbo districts of the Kaffa zone, which are among the 13 districts in the zone.

The study areas are 374 kilometers (Decha) and 330 kilometers (Gimbo) Southwest of Addis Ababa, Ethiopia's capital city. According to the Central Statistics Agency (CSA) of Ethiopia of the year 2007, these districts have a total population of 128,887 people, with 64,438 men and 64,449 women (Decha) and 89,892 people, with 44,774 men and 45,118 women (Gimbo).

### 2.2. Study Design

A health facility-based retrospective study was conducted on secondary data collected from four health centers in selected districts of the zone to assess the five-year (2017-2021) trend of malaria cases.

### 2.3. Source of Information

The malaria laboratory registers logbook of the available health facilities (four health centers) that provide malaria diagnosis and, treatment was included in the study as a source of information. The health centers all use the same type of malaria laboratory register, so the variables captured are the same regardless of where the health centers are located.

### 2.4. Data Collection Methods for Malaria Trend Analysis

The data were transferred from the registers to the checklists that had been prepared before the start of the study. The study variables included were the date of diagnosis, the patient's address, gender, age, the diagnostic tools used, the diagnosis result, and the parasite species identified.

### 2.5. Data Quality Control

Records that were missing one of the above variables were not included in the analysis. Data collectors and supervisors were trained for two days before the study to ensure data quality. They were also taught about the study's data collection tools, variables of interest, rationale, objective, and significance. The principal investigators monitored the entire process on a daily basis, including data collection and entry, to ensure accuracy and consistency.

### 2.6. Data Analysis

Data were entered into an Excel spreadsheet and exported to SPSS version 25.0 for analysis. Seasons, years, gender, age, and malaria parasite species were all used to show trends in malaria transmission using descriptive statistics. Percentages, tables, and figures were used to present the data. A *P*-value less than 0.05 was considered to have statistical significance. The malaria incidence rate was estimated per 1000 person-years, with the total number of person-years as the denominator and the number of new cases as the numerator with the following formula:

(1) $$\begin{array}{c} Incidence\;Rate:\frac{Number\;of\;New\;Cases\;of\;Disease\;or\;Injury\;du\;ring\;Specifie\;d\;Period}{Time\;Each\;Person\;was\;Observe\;d,Totale\;d\;for\;all\;Persons}\text{.} \end{array}$$

### 2.7. Operational Definition

  Malaria prevalence: total number of confirmed cases during a specified period of time × 100 per total population at risk during the same period.
  A confirmed malaria case has been confirmed by a diagnostic test (microscopy and rapid diagnostic test).
  Slide positivity rate (SPR): the proportion of RDT and microscopy slides found positive among the slides examined.
  Population at risk: population living in a geographical area in which locally acquired malaria cases occurred in the current and/or previous years.
  Annual parasite incidence (API): total number of positive slides for malaria parasite in a year × 1000 per total population at risk.

## 3. Results

### 3.1. Prevalence of Malaria in Relation to Demographic Variables

Malaria was more prevalent among adults aged 15 and above, and children under the age of 15 accounted for 5.6% of the total. Regarding the gender distribution, more than half of the screened individuals were females, contributing more to the disease prevalence. Almost two-thirds of the individuals screened for malaria were rural residents, while their counterparts were urban dwellers [Table 1].

### 3.2. Five-Year Trends of Malaria

**Figure 1:**
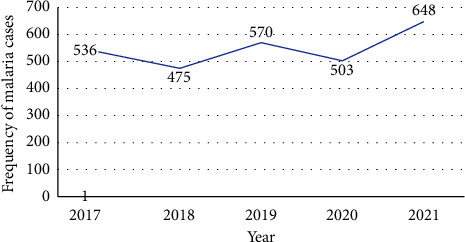
Trend of malaria cases by year at the selected health centers, 2021.

The trend of malaria prevalence was assessed by reviewing the laboratory records and registers of the four health centers, namely, Awurada ketema, Bobagecha, Lalamba, and Ufa. Over five years, of the total 20,045 individuals screened for malaria, *Plasmodium* species were detected in 13.6% (2,732) of them. Among the total number of individuals tested, *P. falciparum*, *P. vivax*, and mixed infections (*P. falciparum* + *vivax*) were detected in 1200 (5.9%), 1114 (5.5%), and 418 (2.1%) of the individuals, respectively. The malaria cases recorded in 2017 were slightly higher than those of the next year but with fewer cases in 2019. The difference lowered to 503 in 2020, which then peaks to its highest over the five years in 2021[Figure 1].

**Figure 2:**
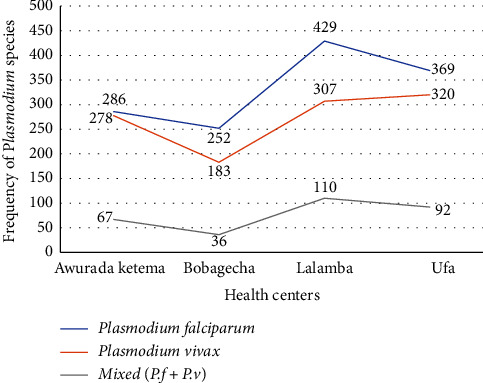
Plasmodium species distribution by health center over the past five years (2017-2021).

Based on their records and registration books, *Plasmodium falciparum* was the most common parasite in all of the health centers included, followed by *Plasmodium vivax*. The Lalamba health center had the most *Plasmodium falciparum* cases, while the Ufa health center in Gimbo had the most *Plasmodium vivax* cases [Figure 2].

## 4. Annual Trends of Malaria Cases

**Figure 3:**
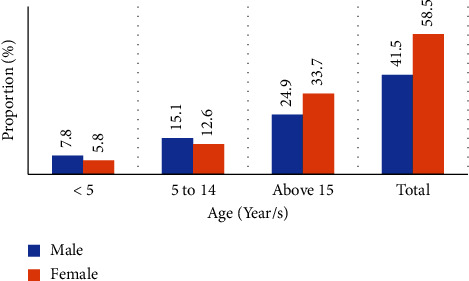
Proportion of malaria cases by age and sex in Decha and Gimbo districts, Southwest Ethiopia (2017-2021).

Of all the total confirmed cases, 1,602 were contributed by patients aged 15 years and above, while 758 (27.8%) and 372 (13.6%) were children between the ages of 5 and 14 and under 5 years, respectively. In total, 41.4% of the population was under the age of 15 years, and the gender distribution with respect to confirmed cases was 1,134 and 1,598 cases for male and female patients, respectively. Overall negativity and positivity rates in the five-year period were 15.2% (1,174) and 12.6% (1,558), respectively. Males had a higher proportion of childhood malaria than females, and the reverse was true in adults [Figure 3].

Between 2017 and 2021, the number of cases increased, with year-to-year variations. There was a noticeable decline in cases from 2017 to 2018 in addition to the rising pattern during the years of 2018 and 2020, which then declined in the years of 2019 and 2021. Confirmed cases fluctuated over the last five years, as noted by the trends. Overall positivity decreased from 13.4% in 2017 to 12.8% in 2018, then increased to 14.2% in 2019, then declined again to 13.7% in 2020, before rising to 14.0% in 2021. Furthermore, between 2018 and 2020, the number of tested individuals and confirmed cases decreased, which was also similar to the rate of positivity [Table 2].

### 4.1. Malaria Incidence Rate

**Figure 4:**
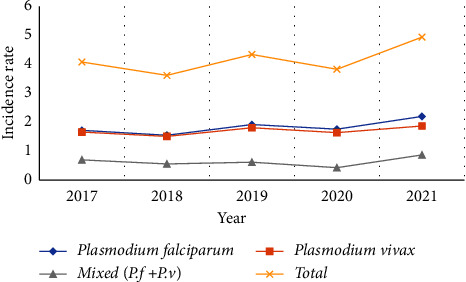
Malaria incidence rate per total population at risk in Decha and Gimbo districts, Southwest Ethiopia (2017-2021).

With an interannual variation, overall malaria incidence decreased from 4.08 in 2017 per 1000 person-years to 3.62 in 2018, then increased to 4.94 in 2021 per 1000 person-years. During the first four years of 2017 to 2020, the incidence rate was almost similar (3.83-4.08) cases per 1000 person-years, but it rose again in 2021 to 4.94 per 1000 person-years. During the same period, the number of screened individuals increased. As a result, the positivity rate decreased and increased at the same time as the number of tested individuals, from 13.4% to 13.6%. During 2018 and 2020, the number of screened individuals, confirmed cases, and the positivity rate all decreased [Figure 4].

### 4.2. Malaria Incidence Rate by Age Groups

**Figure 5:**
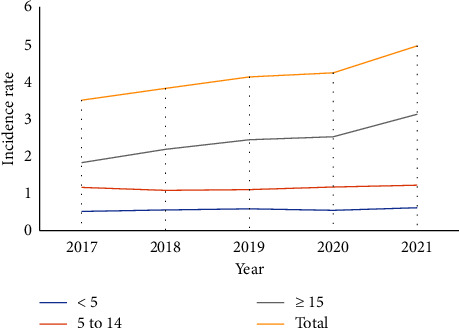
Incidence rate per total population at risk by age group in Decha and Gimbo districts, Southwest Ethiopia (2017-2021).

The incidence of malaria in children under the age of five and those aged 5 to 14 years has remained nearly constant over the last five years. However, there has been an increase in the incidence of malaria in patients over the age of 15 (1.84-3.15) per 1000 person-years (2017-2021). Similarly, the overall malaria incidence rate increased from 3.53 cases per 1000 person-years in 2017 to 5.0 cases per 1000 person-years in 2021 [Figure 5].

### 4.3. Seasonal Distribution of *Plasmodium* Species

In January, February, March, July, and August, malaria cases were lower than the average in the study. Malaria transmission was highest in September, October, and November, with the lowest transmission in January. During the study period, both *P. falciparum* and *P. vivax* were reported in all months. *Plasmodium falciparum* was most prevalent in September, October, and November, accounting for 35.1% of all *P. falciparum* cases. *P. vivax*, on the other hand, peaked in September, October, and November, accounting for 32.7% of the total *P. vivax* reported.

## 5. Discussion

In this malaria record review, which involves all available data from 2017-2021 with complete information, the trends in malaria over the last five years showed a slight decrease in 2018 and 2020 and a slight increase in 2019 and 2021. A light microscope was used to test 61.6% of the total individuals screened for malaria in the four health centers, while an RDT was used to screen the remaining 38.4%. More than half of all the confirmed cases were adults aged 15 and above. Children aged 5-14 and those under 5 years accounted for 27.8% and 13.6% of the remaining confirmed cases, respectively. More than one-third of the malaria cases were in children under the age of 15. In terms of gender distribution, females accounted for more than half of all confirmed cases, while males accounted for 41.5%. Concerning childhood malaria, males had a higher proportion than females, and the reverse was true in adults.

From 2017 to 2021, both the number of individuals screened and confirmed cases increased, with little year-to-year fluctuation. During the first four years from 2017 to 2020, the incidence rate was almost similar (3.83-4.08), but it increased during 2021 to 4.94 per total population at risk. Regarding seasonal variations, malaria transmission was at its highest in the months of September, October, and November in the study areas, with the lowest recorded in January.

The two common forms of the malaria parasite, *P. falciparum* and *P. vivax*, were reported in all months during the study period. *Plasmodium falciparum* peaked in September, October, and November, where 35.1% of the overall *P. falciparum* was reported. Likewise, *P. vivax* peaked during the same months (September, October, and November), with 32.7% of total *P. vivax* reported during the same periods.

In the record review, we found a total of 20, 045 individuals tested with both microscopy and RDT, of which 2,732 records were found with confirmed malaria cases.

However, given that the data came from only four health centers, and the possibility that some of the residents visited private health facilities for fever treatment and malaria diagnosis, a conclusion based solely on this finding may not be credible. Moreover, the information recorded in the health centers was limited to demographic profiles and results of the patients only. As was the case with all public health facilities in Ethiopia, no history of travel before arriving at the health facility could be obtained. In areas where malaria transmission is low, travel to malaria-endemic areas could be a significant risk factor [26, 27].

Therefore, the result of this study (malaria prevalence recorded) was lower than that of other studies from different parts of the world: a six-year malaria trend analysis from Morogoro Rural District, Eastern Tanzania, which employs similar testing methods to our study, revealed 46.19% (35,386/76,604) positive cases [28]. Another seven and five-year malaria trend analysis from Dembia district in two health centers and Dembecha Health Center West Gojjam Zone, Ethiopia showed 21.8% (2,590/11,879) and 16.34% (2086/12,766) malaria confirmed cases, respectively [29, 30].

Additionally, malaria trend analysis of an eight-year period in Boricha District, Southern Ethiopia, found a prevalence of 21.8% (29,554/135,607) [31], a four-year Greater Mekong Subregion China with 19.7% (6175/31,3260) confirmed cases [32], a ten-year trend analysis at Arjo-Didessa, Southwest Ethiopia, revealed a prevalence of 33.4% (18,049/54,020), a ten-year trend analysis at Arjo-Didessa, Southwest Ethiopia, revealed a prevalence of 33.4% (18,049/54,020), a ten-year trend analysis at Arjo-Didessa, Southwest Ethiopia, revealed a prevalence of 33.4% (18,049/54,020) [33], an eight-year malaria trend from Bale Zone, Ethiopia with confirmed cases of 66.7% (10,986/16,465) [34], a three-year trend analysis from East Wollega Zone districts with 21.2% (26,679/125,917) [35] malaria cases, a ten-year trend of malaria prevalence in Asendabo Health Center's Jimma zone, Southwest Ethiopia, with a malaria prevalence of 20.7% (13,624/68,421) [36], and a five-year trend from Guba district, Benishangul-Gumuz regional state, western Ethiopia, revealed a malaria prevalence of 51.04% (8658/16,964) [37] were among malaria trend studies with variable proportions of malaria confirmed cases, all of which were higher than our study.

The abovementioned variations might be due to differences in the number of records incorporated into the review (sample size) and possibly geographical variations. But, some studies revealed a lower malaria prevalence than that of our study. A five-year trend analysis conducted in Ataye, North Shewa, Ethiopia between 2013 and 2017 showed a malaria prevalence of 8.4% (2670/31,810) [38]. Moreover, a five-year and seven-year trend analysis revealed a malaria prevalence of 4.2% (793/19,106) [39] and 6.96% (5010/71,986) [40] from Mojo Town, Central Ethiopia, and North West Tigray, respectively.

The trend analysis in this study could not fully reveal all of the malaria trends in the study area since some of the people seeking malaria diagnosis and treatment may visit other health facilities other than those included in the study, preferring to go to local private clinics, health posts, and drug stores, and this may make conclusions difficult based only on those records of patients served at the selected health facilities.

Moreover, the diagnostic methods used in this study were microscopy and RDTs, which are known for their inferior performance compared to advanced molecular techniques like polymerase chain reaction (PCR) and loop-mediated isothermal amplification (LAMP), which are known to produce highly sensitive and specific results.

### 5.1. Limitation of the Study

The data used in this trend analysis were obtained from secondary sources (laboratory registers), which can affect both the quality of data obtained (*Plasmodium* species identification requires expertise/skilled laboratory technician in malaria blood film microscopy) and data completeness issues, making conclusions difficult based solely on records of patients served at the selected health facilities.

## 6. Conclusion

The malaria trend over the last five years has revealed a nonconsistent pattern of cases in different years. Cases due to malaria have shown an increase in number over the past five years (2017 to 2021). Interannual and intra-annual variations have been observed in the pattern of malaria transmission, as well as inconsistencies in disease burden distribution across age groups and gender. Understanding the temporal and spatial distribution of disease is critical for effective interventional planning.

### 6.1. Recommendation

Although light microscopy and RDTs detected a large number of malaria infections, highly sensitive molecular diagnostic methods such as PCR and LAMP could detect significant number of malaria infections which may not be detected by the less sensitive techniques. As a result, more research is needed to get a better understanding of the malaria trends in the study areas. Regular checks for completeness should be performed by health facilities to improve the quality and consistency of their laboratory records and logbooks.

## Figures and Tables

**Table 1:** Prevalence of malaria in relation to demographic variables from record review.

Variable	Category	Individuals with no confirmed cases *n* (%)	Individuals with confirmed cases *n* (%)	Total *n* (%)
Sex	Male	7,188 (35.8)	1,134 (5.7)	8,322 (41.5)
	Female	10,130 (50.5)	1,598 (7.9)	11,728 (58.5)

Age in year/s	<5	1,754 (8.7)	372 (1.8)	2,126 (10.6)
	5-14	5,420 (27.0)	758 (3.8)	6,178 (30.8)
	≥15	10,139 (50.6)	1,602 (8.0)	11,741 (58.6)

Residence	Urban	6,088 (30.4)	1,083 (5.4)	7,171 (35.8)
	Rural	11,235 (56.0)	1,639 (8.2)	12,874 (64.2)

**Table 2:** Slide positivity rates and *Plasmodium* species composition of malaria parasite in Decha and Gimbo districts, Southwest Ethiopia (2017-2021).

Year	Total suspected cases	Positivity rate *n* (%)			
		Total	*Plasmodium falciparum*	*Plasmodium vivax*	Mixed (P.*f* + *P*.v)
2017	4009	536 (13.4)	226 (5.6)	218 (5.4)	92 (2.3)
2018	3706	475 (12.8)	203 (5.5)	198 (5.3)	74 (2.0)
2019	4011	570 (14.2)	252 (6.3)	237 (5.9)	81 (2.0)
2020	3677	503 (13.7)	231 (6.3)	215 (5.9)	57 (1.6)
2021	4642	648 (14.0)	288 (6.2)	246 (5.3)	114 (2.5)
Total	20,045	2,732 (13.6)	1200 (6.0)	1,114 (5.6)	418 (2.1)

## Data Availability

On reasonable request, the data related to this study can be obtained from the corresponding author.

## Abbreviations

CSA: Central Statistics Agency
IRS: Indoor residual spraying
LAMP: Loop-mediated isothermal amplification
LLIN: Long-lasting insecticidal net
LM: Light microscopy
PCR: Polymerase chain reaction
RBC: Red blood cell
RDT: Rapid diagnostic test
WHO: World Health Organization.
